# Life-Threatening Hypercalcemia Revealing Diffuse and Isolated Acute Sarcoid-Like Myositis: A New Entity? (A Case-Series)

**DOI:** 10.1097/MD.0000000000003089

**Published:** 2016-03-11

**Authors:** Arthur Mageau, Aude Rigolet, Khadija Benali, Maria Chauchard, Salima Ladjeroud, Isabelle Mahe, Thierry Maisonobe, Marie-Paule Chauveheid, Thomas Papo, Karim Sacre

**Affiliations:** From the Département de Médecine Interne, Hôpital Bichat, Université Paris Diderot, PRES Sorbonne Paris Cité, Assistance Publique Hôpitaux de Paris (AM, MPC, TP, KS); Département de Médecine Interne, Centre de référence de pathologie neuromusculaire, Hôpital Pitié-Salpêtrière, Université Pierre et Marie Curie, Assistance Publique Hôpitaux de Paris (AR); Département de Médecine Nucléaire (KB); Département de Médecine Interne, Hôpital Saint Antoine, Université Pierre et Marie Curie, Assistance Publique Hôpitaux de Paris (MC); Département de Radiologie (SL), Hôpital Bichat, Université Paris Diderot, PRES Sorbonne Paris Cité, Assistance Publique Hôpitaux de Paris; Département de Médecine Interne, Hôpital Louis Mourier, Université Paris Diderot, PRES Sorbonne Paris Cité, Assistance Publique Hôpitaux de Paris, EA REMES 7334 Recherche Clinique ville-hôpital, Méthodologies et Société (IM); Département de Neuropathologie et Neurophysiologie, Hôpital Pitié-Salpêtrière, Université Pierre et Marie Curie, Assistance Publique Hôpitaux de Paris (TM); Département Hospitalo-Universitaire FIRE (Fibrosis, Inflammation and Remodelling in Renal and Respiratory Diseases), Université Paris Diderot, PRES Sorbonne Paris Cité (TP, KS); and INSERM U1149, Université Paris Diderot, Laboratoire d’excellence INFLAMEX, PRES Sorbonne Paris Cité (TP, KS), Paris, France.

## Abstract

Up to 50% patients with sarcoidosis display extra-pulmonary disease. However, initial and isolated (ie, without lung disease) acute muscular involvement associated with pseudo-malignant hypercalcemia is very uncommon. We report on 3 cases of life-threatening hypercalcemia revealing florid and isolated acute sarcoid-like myositis.

All patients complained of fatigue, progressive general muscle weakness, and weight loss. Laboratory tests showed a severe life-threatening hypercalcemia (>3.4 mmol/L). Hypercalcemia was associated with increased serum level of 1,25-(OH)_2_ vitamin D and complicated with acute renal failure. One patient displayed acute pancreatitis due to hypercalcemia.

In all cases, PET-scan, performed for malignancy screening, incidentally revealed an intense, diffuse, and isolated muscular fluorodeoxyglucose (FDG) uptake consistent with diffuse non-necrotizing giant cells granulomatous myositis demonstrated by muscle biopsy. Of note, creatine phosphokinase blood level was normal in all cases. No patients displayed the usual thoracic features of sarcoidosis.

All patients were treated with high dose steroids and achieved rapid, complete, and sustained remission. A review of English and French publications in Medline revealed 5 similar published cases.

Steroid-sensitive acute sarcoid-like myositis causing high calcitriol levels and life-threatening hypercalcemia should be recognized as a separate entity.

## INTRODUCTION

Sarcoidosis is a multi-system granulomatous disease of unknown etiology. Although muscle involvement is described in up to 80% of patients with sarcoidosis,^1^ clinically significant myositis is rare.^2,3^ In such setting, acute myositis may even be less frequent.^4,5^ In addition, muscular sarcoidosis typically occurs in patients with already known sarcoidosis.^1,6^ Eventually, 90% of patients with extra-pulmonary sarcoidosis have lung involvement. Hence, isolated muscular sarcoidoisis is exceedingly rare.^3^

Calcitriol-mediated hypercalcemia is a well-known complication of sarcoidosis. Mild to severe hypercalcemia, however, is detected in less than 5% of patients and pseudo-malignant hypercalcemia (>3.5 mmol/L) is very unusual.^2,3,7,8^

Here, we report on 3 cases of life-threatening hypercalcemia that revealed florid and isolated acute sarcoid-like myositis. We review the literature and analyzed 5 other observations related to this very unusual disorder.

## METHODS

### Patients

The study included 3 patients who were followed in Internal Medicine departments from 3 French university hospitals (Bichat Hospital, Louis Mourier Hospital, and Pitie-Salpêtrière Hospital, Assistance Publique Hôpitaux de Paris) between September 2010 and November 2013. Demographic, medical history, laboratory, imaging, histology, treatment, and follow-up data were extracted from medical records.

### Ethical Statement

Our study is a retrospective human non-interventional study. According to the Public Health French Law (art L 1121–1–1, art L 1121–1–2), approval from institutional review board and written consent are not required for human non-interventional studies. For ethical consideration, patients were, however, informed that data that were collected in medical records might be used for research study in accordance with privacy rule. The study protocol conforms to the ethical guidelines of the 1975 Declaration of Helsinki.

### Literature Review

MEDLINE (National Library of Medicine, Bethesda, MD) search was performed until August 2015 using [sarcoid-like myositis] OR [granulomatous myositis] OR [muscular granulomatosis] OR [muscular sarcoidosis] OR [myositis] AND [hypercalcemia] items. Analysis was limited to article published in English and French. Fifteen cases were identified. Eight cases were excluded because of irrelevant (n = 3)^9,10^ or incomplete (n = 5) information regarding granulomatous disease or serum calcemia.^4,11–13^ Two cases were excluded because of language issue (n = 2).^14,15^

## RESULTS

### Case n°1

A 34-year-old non-smoker man went to the emergency room (ER) for abdominal pain. He had no significant past medical history and did not take any treatment. He complained of fatigue, general muscle weakness, and weight loss over a few weeks. Physical examination was normal. C-reactive protein was measured at 23 mg/L. Blood creatinine was 214 μmol/L. Calcium blood level was 3.43 mmol/L. Serum lipase level was more than 20-fold the upper normal limit. Hemogram and liver tests were normal. The diagnosis of acute pancreatitis caused by hypercalcemia was considered and the patient was hospitalized.

Further, serum testing showed that serum levels of parathormon (PTH), parathyroid hormone-related protein (PTH-rp), and 25-OH vitamin D were low while 1,25-(OH)_2_ vitamin D level was high. Blood level of angiotensin-converting enzyme (ACE) was slightly increased. Serum creatine phosphokinase level and protein electrophoresis were normal.

Abdominal ultrasonography only detected renal stones without parenchymal calcification.

**FIGURE 1:**
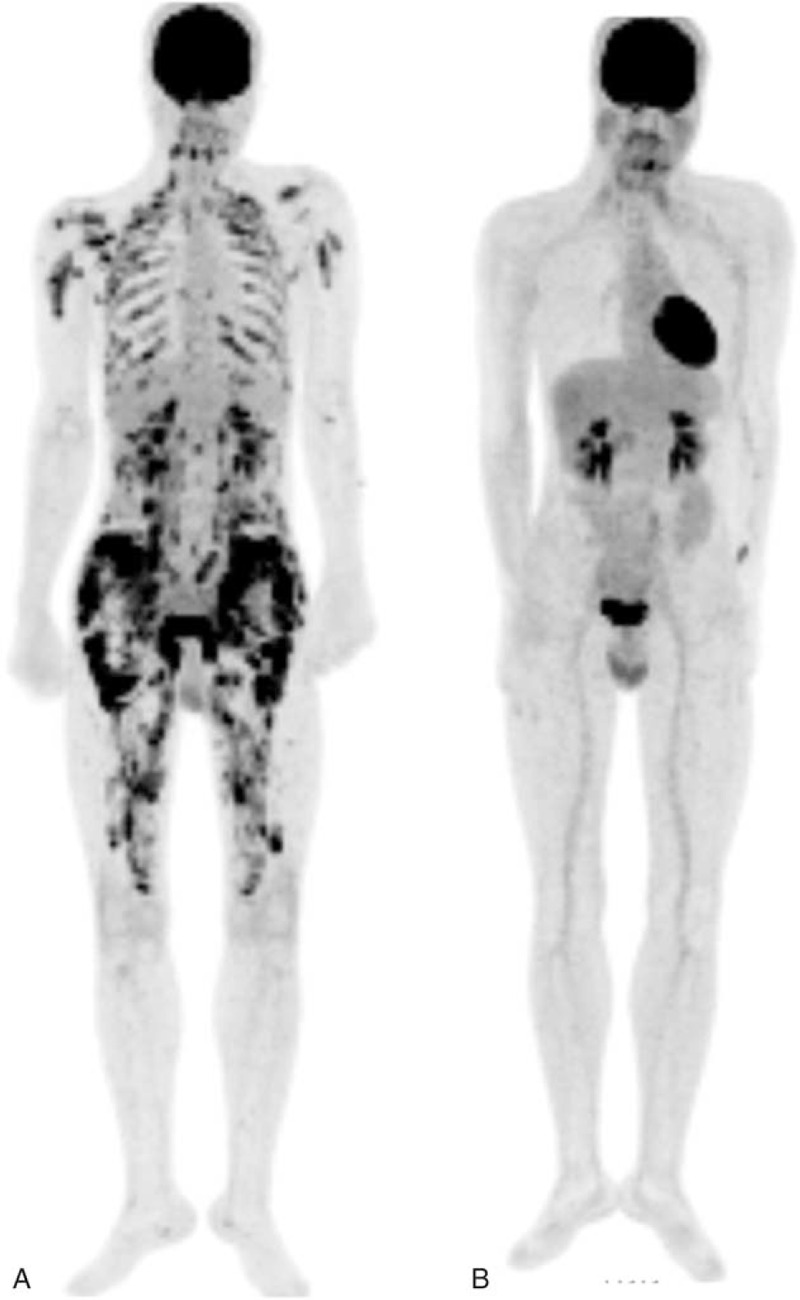
PET-scan findings, (A) maximum intensity projection coronal images revealing multiple linear and nodular intense FDG uptakes in intercostal, back, shoulder, and thigh muscles. (B) Complete regression of aforementioned pathologic FDG uptakes after 2 months of steroid treatment.

**FIGURE 2:**
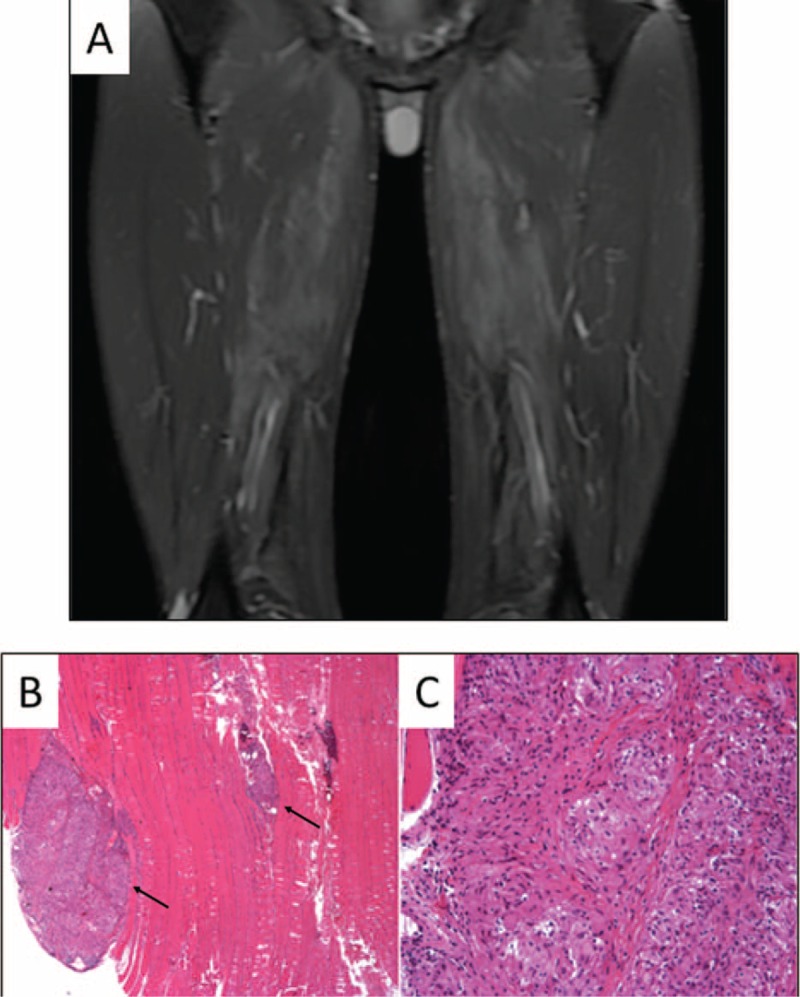
MRI and deltoid muscle biopsy findings, (A) coronal STIR weighted images showing a high signal intensity of the following muscles: gluteus maximus, adductor brevis, adductor magnus, semi-membranosus, and lateral portion of the biceps femoris. (B) Paraffin-embedded longitudinal section with haematoxylin-eosin stains showed two (arrows) intra muscular non-necrotizing granuloma (270×). (C) Large collection of epithelioid histiocytes cells with small lymphocytic cells in the periphery were noted (540×).

A PET-scan was performed and showed an unexpected, intense, diffuse, and isolated muscular fluorodeoxyglucose (FDG) uptake (Figure 1A). Muscle Magnetic Resonance Imaging (MRI) showed bilateral and symmetric abnormal signal intensity in the proximal thigh (Figure 2A). Of note, electromyography was normal.

Deltoid muscle biopsy showed numerous intra muscular non-necrotizing giant cells granuloma (Figure 2B and C). Chest-CT scan and pulmonary function testing were normal. Bronchoscopy with bronchial biopsy and bronchoalveolar lavage did not revealed granuloma or alveolitis.

The diagnosis of severe hypercalcemia caused by florid and isolated sarcoid-like myositis was made. High-dose steroid treatment was initiated. Blood calcium and creatinine levels normalized in several days. After 2 months of treatment, PET-scan (Figure 1B) and muscle MRI confirmed a full remission. The patient was still receiving prednisone at daily dose of 9 mg, 21 months after diagnosis. No relapse occurred during follow-up.

### Case n°2

A 50-year-old man without past medical history went to the ER for fatigue, general muscle weakness, and weight loss. She denied tobacco use and was treated with esomeprazole for recurrent pyrosis. Physical examination was normal with 5/5 proximal and distal muscle strength. Standard biology test revealed a calcium blood level of 3.81 mmol/L. Blood creatinine was 163 μmol/L. Complete blood cell count, electrolytes, C-reactive protein, creatine phosphokinase, and liver tests were normal. Serum level of PTH was low. A PET scan performed for occult malignancy screening revealed an isolated, diffuse, and intense FDG uptake in muscles. Electromyography was not performed. Muscle biopsy specimen obtained from the right calf showed numerous intra muscular non-necrotizing giant cells granuloma. Intravenous rehydration, bisphosphonates, and steroids were prescribed. Blood calcium and creatinine levels promptly normalized in several days. Unfortunately, the patient was lost to follow-up soon after discharge.

### Case n°3

A 71-year-old non-smoking woman was referred for asthenia, weight loss, and increasing painful proximal leg and arm weakness over a 2-week period. She had a past history of hypothyroidism, high blood pressure, dyslipidemia, and essential thrombocythemia. Treatment included lercanidipin, levothyroxine, and low dose aspirin. Physical examination was normal. No muscle tenderness or atrophy was noted. Blood screening showed a mild isolated anemia (Hb 9.8 g/dL). Blood calcium level was 3.9 mmol/L. Serum creatinine was 243 μmol/L. C-reactive protein, creatinine phosphokinase, liver tests, and thyroid function tests were normal. PTH serum level was low. Serum protein electrophoresis revealed a monoclonal IgG lambda protein measured at 3 g/L. Blood level of ACE and 1,25-(OH)_2_ vitamin D were around 2-fold the upper normal limit. A PET scan was performed for malignancy screening and showed isolated, intense, and diffuse FDG uptake of skeletal muscles. Scare perilymphatic micronodules were noted on chest CT-scan without mediastinal nodal enlargement. A deltoid muscle biopsy specimen revealed several diffuse, large, and non-necrotizing giant cells granuloma. The bone marrow smear analysis showed no abnormal plasma cells. Treatment with prednisone, 45 mg daily and methotrexate (MTX), 15 mg weekly was started. The patient reported prompt clinical improvement. Blood calcium and creatinine normalized in several days. Four months later, MTX was switched for mycophenolate mofetil (MMF) because of stomatitis while the patient was in complete remission. PET-scan control performed at 6 months of treatment normalized. Thirty months later, she remains well on low dose prednisone and MMF.

### Literature Review

To our best knowledge, 8 cases of life-threatening hypercalcemia revealing isolated acute sarcoid-like myositis, including our 3 cases, have been reported so far.^16–20^Table 1 summarizes the characteristics of these patients. Median age was 51.5 (range: 34–75) years. Sex ratio (F/M) was 1.7/1. All patients suffered severe hypercalcemia ranging from 3.34 to 5.02 mmol/L complicated with renal failure. Progressive weakness was reported in all cases (8/8) contrasting with normal (5/8), and painless (6/8) muscle strength. CPK blood level was always normal (8/8). Electromyogram showed a myopathic pattern (3/4). PET-scan revealed an intense, diffuse, and isolated muscular FDG uptake (5/5). In all cases, PET-scan was performed for malignancy screening and incidentally revealed the muscle disease. Muscle biopsy showed diffuse non-necrotizing granulomas in all cases. Of note, thoracic features of sarcoïdosis (ie, bilateral hilar and mediastinal nodal enlargement and lung micronodules with a perilymphatic distribution) were absent in all but one case. All patients received corticosteroids. Immunosuppressive drugs were prescribed in two cases. All but one patient achieved rapid, complete, and sustained remission under treatment with a median follow-up of 18.5 (range: 3–24) months. One patient died from acute congestive heart failure with autopsy revealing a disseminated giant cell granulomatous process affecting skeletal, cardiac, and gastrointestinal smooth muscle.^17^ Relapse occurred in only one case.^20^

**TABLE 1:**
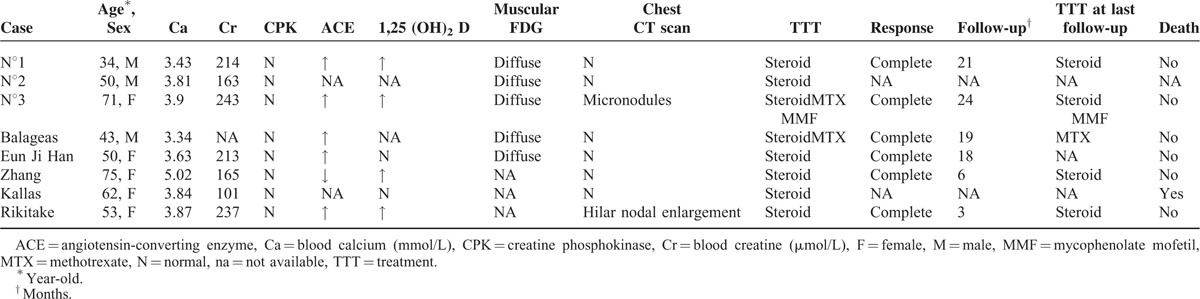
Characteristic of Studied Patients and Literature Review

## DISCUSSION

Mild to severe hypercalcemia is detected in less than 5% of patients and hypercalcemia requiring emergency treatment as reported here is very uncommon.^3,7,8^ The hypercalcemia in sarcoidosis is mostly the result of increased absorption of intestinal calcium due to increased 1,25-(OH)_2_ vitamin D production by granuloma.^8,17,21^ In our study, the elevated (or inappropriately normal) 1,25-(OH)_2_ vitamin D associated with suppressed PTH pointed to the presence of excessive 1-hydroxylase activity by sarcoid macrophages. Accordingly, treatment with prednisone led to dramatic clinical improvement. Sarcoid macrophages have been reported to produce PTH-rp that may contribute to hypercalcemia as well.^22,23^ In our series, PTH-rp was measured in only one case but was undetectable.

Asymptomatic muscle involvement is common in systemic sarcoidosis, with about 50% to 80% of routine muscle biopsies showing abnormalities.^6^ Muscle symptoms are, however, only observed in less than 0.5% of cases and are rarely inaugural.^2,3^ In our cases, the pattern of muscular involvement did not fit with any of the 3 classical—chronic, nodular, and acute-myositis types described in patients with sarcoidosis.^1,4,5,24–26^ Indeed, the course of chronic sarcoid myositis—the most common pattern—is characteristically slow and steadily progressive, with a limited benefit of steroid treatment. The nodular type of sarcoid muscle involvement is characterized by palpable nodules that do not lead to weakness or functional disability. The acute sarcoid myositis mimics the presentation of acute polymyositis with elevated CPK blood levels. Moreover, muscle involvement in classic systemic sarcoidosis is not known to be associated with severe hypercalcemia (Table 2).

**TABLE 2:**
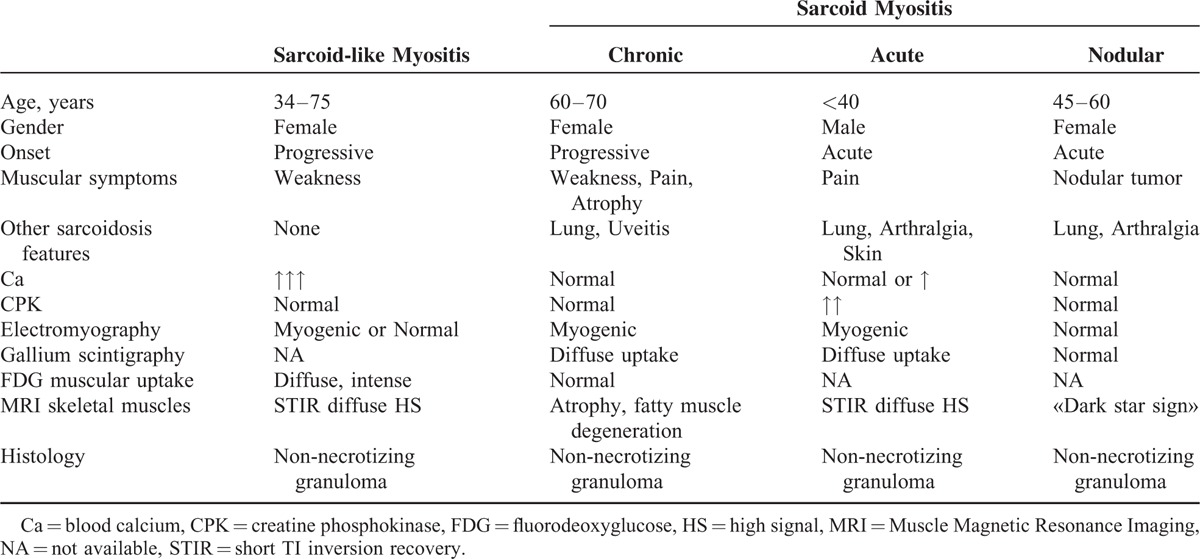
Sarcoid and Sarcoid-like Myositis: Differing Patterns^6,27–33^

Sarcoid lung involvement occurs in 80% to 90% of patients with extra-pulmonary sarcoidosis. A typical sarcoidosis presentation with uncommon locations to a single organ and no lung involvement is clearly challenging.^2,3^

Our series highlights the high diagnostic yield of PET-scan in such setting. In most cases, muscular involvement, despite its extensive pattern, was not clinically suspected because of unspecific symptoms and normal CPK. Only PET-scan—that was performed to disclose underlying malignancy in a context of profound hypercalcemia—clearly pointed to a diffuse and active muscle inflammation. Moreover, PET-scan appeared useful for monitoring the treatment response.

In conclusion, the isolated granulomatous myositis causing high calcitriol levels should be considered a separate entity, distinct from systemic sarcoidosis. Severe hypercalcemia revealing a diffuse granulomatous disorder limited to muscles might be life threatening and appears highly steroid-sensitive.

## References

[1] Silverstein A Siltzbach LE Muscle involvement in sarcoidosis. Asymptomatic, myositis, and myopathy. *Arch Neurol* 1969; 21:235–241. 5802453 10.1001/archneur.1969.00480150025002

[2] Baughman RP Teirstein AS Judson MA Clinical characteristics of patients in a case control study of sarcoidosis. *Am J Respir Crit Care Med* 2001; 164:1885–1889. 11734441 10.1164/ajrccm.164.10.2104046

[3] Valeyre D Prasse A Nunes H Sarcoidosis. *Lancet* 2014; 383:1155–1167. 24090799 10.1016/S0140-6736(13)60680-7

[4] Ost D Yeldandi A Cugell D Acute sarcoid myositis with respiratory muscle involvement. Case report and review of the literature. *Chest* 1995; 107:879–882. 7874972 10.1378/chest.107.3.879

[5] Wolfe SM Pinals RS Aelion JA Myopathy in sarcoidosis: clinical and pathologic study of four cases and review of the literature. *Semin Arthritis Rheum* 1987; 16:300–306. 3299715 10.1016/0049-0172(87)90008-4

[6] Fayad F Duet M Orcel P Systemic sarcoidosis: the “leopard-man” sign. *Joint Bone Spine* 2006; 73:109–112. 16256397 10.1016/j.jbspin.2005.04.007

[7] Conron M Young C Beynon HL Calcium metabolism in sarcoidosis and its clinical implications. *Rheumatology (Oxford)* 2000; 39:707–713. 10908687 10.1093/rheumatology/39.7.707

[8] Sharma OP Vitamin D, calcium, and sarcoidosis. *Chest* 1996; 109:535–539. 8620732 10.1378/chest.109.2.535

[9] Yamada S Taniguchi M Tsuruya K Recurrent sarcoidosis with psoas muscle granuloma and hypercalcaemia in a patient on chronic haemodialysis. *Nephrology (Carlton)* 2009; 14:452–453. 19563388 10.1111/j.1440-1797.2009.01097.x

[10] Mozaffar T Lopate G Pestronk A Clinical correlates of granulomas in muscle. *J Neurol* 1998; 245:519–524. 9747915 10.1007/s004150050236

[11] Le Roux K Streichenberger N Vial C Granulomatous myositis: a clinical study of thirteen cases. *Muscle Nerve* 2007; 35:171–177. 17068767 10.1002/mus.20683

[12] Boon ES Cozijn D Brombacher PJ Enhanced production of calcitriol, and hypercalcaemia in a patient with sarcoidosis provoked by daily intake of calciol. *Eur J Clin Chem Clin Biochem* 1993; 31:679–681. 8292670

[13] Hermanrud T Janner JH Steffensen IE Hypercalcaemia can be the only initial symptom of sarcoidosis. *Ugeskr Laeger* 2014; 176:9. 25096565

[14] Sugiura A Noshiro H Ieiri N Muscular sarcoidosis associated with acute renal failure due to hypercalcemia. *Nihon Naika Gakkai Zasshi* 2003; 92:2404–2406. 14743758 10.2169/naika.92.2404

[15] Shimizu S Fukase M Fujita T A case of muscular sarcoidosis showing coexistence of hypercalcemia and renal insufficiency. *Nihon Naika Gakkai Zasshi* 1985; 74:473–479. 4045281 10.2169/naika.74.473

[16] Rikitake Y Kinoshita Y Kotani Y Sarcoidosis with hypercalcemia–successful treatment of renal insufficiency and renal calcification with prednisolone. *Intern Med* 1994; 33:222–225. 8069017 10.2169/internalmedicine.33.222

[17] Kallas M Green F Hewison M Rare causes of calcitriol-mediated hypercalcemia: a case report and literature review. *J Clin Endocrinol Metab* 2010; 95:3111–3117. 20427501 10.1210/jc.2009-2673

[18] Zhang JT Chan C Kwun SY A case of severe 1,25-dihydroxyvitamin D-mediated hypercalcemia due to a granulomatous disorder. *J Clin Endocrinol Metab* 2012; 97:2579–2583. 22639294 10.1210/jc.2012-1357

[19] Han EJ Jang YS Lee IS Muscular sarcoidosis detected by F-18 FDG PET/CT in a hypercalcemic patient. *J Korean Med Sci* 2013; 28:1399–1402. 24015050 10.3346/jkms.2013.28.9.1399 PMC3763119

[20] Balageas A Sanguinet F Lequen L Muscular sarcoidosis: a case report of muscle and fascia involvement and literature. *Rev Med Interne* 2013; 34:706–712. 24367812 10.1016/j.revmed.2013.02.008

[21] Sharma OP Hypercalcemia in sarcoidosis. The puzzle finally solved. *Arch Intern Med* 1985; 145:626–627. 3985722

[22] Zeimer HJ Greenaway TM Slavin J Parathyroid-hormone-related protein in sarcoidosis. *Am J Pathol* 1998; 152:17–21. 9422518 PMC1858120

[23] van Raalte DH Goorden SM Kemper EA Sarcoidosis-related hypercalcaemia due to production of parathyroid hormone-related peptide. *BMJ Case Rep* 2015; 2015: 10.1136/bcr-2015-210189 PMC4499713 26160550

[24] Delaney P Neurologic manifestations in sarcoidosis: review of the literature, with a report of 23 cases. *Ann Intern Med* 1977; 87:336–345. 197863 10.7326/0003-4819-87-3-336

[25] Fonseca GA Baca S Altman RD Acute myositis and dermatitis as the initial presentation of sarcoidosis. *Clin Exp Rheumatol* 1993; 11:553–556. 8275593

[26] Jamal MM Cilursu AM Hoffman EL Sarcoidosis presenting as acute myositis. Report and review of the literature. *J Rheumatol* 1988; 15:1868–1871. 3068366

[27] Fayad F Liote F Berenbaum F Muscle involvement in sarcoidosis: a retrospective and followup studies. *J Rheumatol* 2006; 33:98–103. 16395757

[28] Aptel S Lecocq-Teixeira S Olivier P Multimodality evaluation of musculoskeletal sarcoidosis: Imaging findings and literature review. *Diagn Interv Imaging* 2016; 97:5–18. 25883076 10.1016/j.diii.2014.11.038

[29] Marie I Levesque H Manrique A Positron emission tomography in the diagnosis of muscular sarcoidosis. *Am J Med* 2007; 120:e1–e2. 17275433 10.1016/j.amjmed.2006.05.052

[30] Otake S Sarcoidosis involving skeletal muscle: imaging findings and relative value of imaging procedures. *AJR Am J Roentgenol* 1994; 162:369–375. 8310929 10.2214/ajr.162.2.8310929

[31] Kobak S Sarcoidosis: a rheumatologist's perspective. *Ther Adv Musculoskelet Dis* 2015; 7:196–205. 26425148 10.1177/1759720X15591310 PMC4572362

[32] Wieers G Lhommel R Lecouvet F A tiger man. *Lancet* 2012; 380:1859. 22922001 10.1016/S0140-6736(12)60683-7

[33] Maeshima S Koike H Noda S Clinicopathological features of sarcoidosis manifesting as generalized chronic myopathy. *J Neurol* 2015; 262:1035–1045. 25712543 10.1007/s00415-015-7680-0

